# Amoxillin- and pefloxacin-induced cholesterogenesis and phospholipidosis in rat tissues

**DOI:** 10.1186/s12944-015-0011-8

**Published:** 2015-02-21

**Authors:** Solomon O Rotimi, David A Ojo, Olusola A Talabi, Regina N Ugbaja, Elizabeth A Balogun, Oladipo Ademuyiwa

**Affiliations:** Department of Biological Sciences, Covenant University, Ota, Nigeria; Department of Microbiology, Federal University of Agriculture, Abeokuta, Nigeria; Medical Centre, Federal University of Agriculture, Abeokuta, Nigeria; Department of Biochemistry, Federal University of Agriculture, Abeokuta, Nigeria; Department of Biochemistry, University of Ilorin, Ilorin, Nigeria

**Keywords:** Amoxillin, Pefloxacin, Dyslipidemia, Cholesterogenesis, Phospholipidosis

## Abstract

**Background:**

To investigate whether amoxillin and pefloxacin perturb lipid metabolism.

**Methods:**

Rats were treated with therapeutic doses of each antibiotic for 5 and 10 days respectively. Twenty four hours after the last antibiotic treatment and 5 days after antibiotic withdrawal, blood and other tissues (liver, kidney, brain, heart and spleen) were removed from the animals after an overnight fast and analysed for their lipid contents.

**Results:**

Both antibiotics produced various degrees of compartment-specific dyslipidemia in the animals. While plasma and erythrocyte dyslipidemia was characterised by up-regulation of the concentrations of the major lipids (cholesterol, triglycerides, phospholipids and free fatty acids), hepatic and renal dyslipidemia was characterised by cholesterogenesis and phospholipidosis. Splenic dyslipidemia was characterised by cholesterogenesis and decreased phospholipid levels. Cardiac and brain cholesterol contents were not affected by the antibiotics. A transient phospholipidosis was observed in the brain whereas cardiac phospholipids decreased significantly. Lipoprotein abnormalities were reflected as down-regulation of HDL cholesterol. Furthermore, the two antibiotics increased the activity of hepatic HMG-CoA reductase. Although erythrocyte phospholipidosis was resolved 5 days after withdrawing the antibiotics, dyslipidemia observed in other compartments was still not reversible.

**Conclusion:**

Our findings suggest that induction of cholesterogenesis and phospholipidosis might represent additional adverse effects of amoxillin and pefloxacin.

## Introduction

Antimicrobial agents exemplify one of the most dramatic advances of modern medicine. Their discovery, development and clinical use during the 20th century have decreased substantially the morbidity and mortality from bacterial infections once considered incurable and lethal [1]. Their remarkably powerful and specific activity stems from their selectivity for targets that are either unique to the microorganisms or much more important in them as compared to humans.

**Figure 1:**
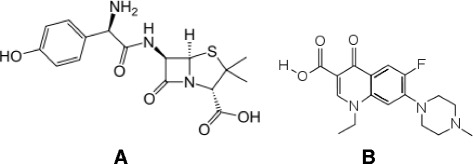
**Chemical structure of amoxillin (A) and pefloxacin (B).**

Amoxillin [(2S, 5R, 6R)-6-{[(2R)-2-amino-2-(4-hydroxyphenyl)-acetyl] amino}3, 3-dimethyl-7-oxo-4-thia-1-azabicyclo [3.2.0] heptane-2-carboxylic acid] and pefloxacin [1-ethyl-6-fluoro-7-(4-methylpiperazin-1-yl)-4-oxoquinoline-3-carboxylic acid] are broad spectrum antimicrobial agents used in the treatment of typhoid infection. While amoxillin is a β-lactam aminopenicillin (Figure 1A), pefloxacin is a fluoroquinolone (Figure 1B). Both are active against gram-negative bacteria. Amoxillin acts by inhibiting the synthesis of bacterial cell walls. It inhibits cross-linkage between the linear peptidoglycan polymer chains that make up a major component of the cell walls of both gram-positive and gram-negative bacteria [2]. Pefloxacin on the other hand owes it antibiotic activity to its blocking of bacterial DNA synthesis [1,3]. It inhibits bacterial topoisomerase II (DNA gyrase) and topoisomerase IV [1,3]. Inhibition of DNA gyrase prevents the relaxation of positively supercoiled DNA that is required for normal transcription and replication. Inhibition of topoisomerase IV interferes with separation of replicated chromosomal DNA into the respective daughter cells during cell division [1,3].

Although amoxillin and pefloxacin are generally considered safe and well tolerated, they have been associated with a wide range of adverse effects [4-7]. Adverse effects for both antibiotics range from fever, nausea, diarrhea and vomiting to major allergic reactions including photo-sensitivity and skin rash [4-7]. Mental changes, light-headedness, confusion and anxiety, have also been reported for both antibiotics [6,8]. Tendinopathy, sometimes resulting in spontaneous rupture of tendons, has also been reported as a side effect of treatment with pefloxacin [7,9,10].

Although these data about amoxillin and pefloxacin have been known for sometime, nothing has been published on the interaction of these antibiotics with lipids. Interest in the interaction of drugs with lipids derives from the observation that lipids play a major role in the pathogenesis and progression of various disease conditions. Furthermore, dyslipidemia is currently becoming a confounding factor not only in new drug development, but also in assessing safety of earlier approved drugs so as to uncouple dyslipidemia from toxic manifestations of the drug. Given these antecedents, the present study was an attempt to investigate the effects of therapeutic doses of amoxillin and pefloxacin on lipid metabolism in the tissues of the rat.

## Materials and methods

### Chemicals

Pefloxacin was a product of Lek Pharmaceutical and Chemical Company, Ljubljana, Slovenia, while amoxillin was obtained from Beecham Pharmaceuticals, Brentford, England. All other chemicals used in this study were of the purest grade available and were obtained from British Drug House (BDH) Chemicals Limited, Poole, England and Sigma-Aldrich, Missouri, U. S. A.

### Animals and treatment

Experimental protocols were conducted in accord with guidelines of the Institutional Animal Care and Use Committee and were approved by the Animal Ethical Committee of the Department of Biochemistry, Federal University of Agriculture, Abeokuta, Nigeria.

Sixty five male albino rats (bred in the animal holding of Nigerian Institute of Medical Research (NIMR), Lagos, Nigeria) with a mean body weight of 230 g were used for the experiment. They were housed in an animal room with normal controlled temperature (22 ± 2°C) and a regular 12 h light–dark cycle (06:00–18:00 h). They were allowed 14 days to acclimatise before the commencement of antibiotic exposure. The animals were maintained on a standard pellet diet.

At the start of experiments (Day 0), 5 animals were sacrificed to obtain baseline data and the remaining animals were divided into 12 groups of 5 animals each (six groups for pefloxacin and six groups for amoxillin). Three groups were treated with either amoxillin (7.14 mg/kg body weight, 8 hourly) or pefloxacin (5.71 mg/kg body weight, 12 hourly) for 5 and 10 days respectively. The antibiotics were constituted in 5% dextrose and were prepared fresh before each administration. They were administered in a total volume of 0.1 ml. Control animals received equivalent volume of 5% dextrose (8 hourly in amoxillin group and 12 hourly in pefloxacin group). All drug administration was by the intraperitoneal route. During the experiment, the animals were allowed free access to food and distilled water.

At the end of the antibiotic treatment and 5 days after the discontinuation of the antibiotics, blood was collected from the animals into heparinised tubes by cardiac puncture under light ether anaesthesia after an overnight fast. Liver, kidney, brain, heart and spleen were removed from the animals for biochemical analyses. Blood samples were centrifuged to separate plasma and red blood cells. All samples were stored at-20°C until analysed.

### Biochemical analyses

#### Plasma and lipoprotein lipid profiles

Determination of the major lipids (cholesterol, triglycerides, phospholipids and free fatty acids) in plasma and lipoproteins followed established procedures. Details of these have been given in our earlier studies [11-16].

#### Organ and erythrocyte lipid profiles

Lipids were extracted from the organs (liver, kidney, heart, lung, brain and spleen) as described by Folch et al. [17] while extraction of erythrocyte lipids followed the procedure described by Rose and Oklander [18]. After washing with 0.05 M KCl solution, aliquots of the lipid extracts were then used for the determination of lipid profiles. Details of these are given as reported earlier [11-16].

#### Isolation of erythrocyte membrane and determination of its lipid profile

Erythrocyte membranes were prepared according to the method described by Hanahan and Ekholm [19]. Briefly, blood samples were centrifuged at 5000 rpm for 15 minutes at 4°C. Plasma and buffy coat were removed by careful suction and the cells were resuspended in isotonic Tris–HCl buffer pH 7.6. After mixing by inversion, the samples were recentrifuged at 5000 rpm for 15 minutes at 4°C. The supernatant was again removed by careful suction and a few red cells were sacrificed to remove any remaining buffy layer. This washing procedure was repeated twice. The washed cells were then suspended in isotonic Tris–HCl buffer pH 7.6 to an approximate hematocrit of 50% and were kept on ice. The samples were mixed gently by inversion for about 1 minute before membrane preparation. 5 ml aliquots of the 50% cell suspensions were transferred to 50 ml polyethylene tubes. Thirty ml of hypotonic Tris–HCl buffer pH 7.6 were added to the cell suspension for osmotic lysis. After allowing the tubes to stand for about 10 minutes, they were centrifuged at 20,000 rpm for 20 minutes at 4°C. The supernatants were discarded and the pellets resuspended in 10 ml Tris–HCl and centrifuged for 20 minutes at 20,000 rpm at 4°C. The pellets were washed four times until the membranes were colourless. Finally, the resultant pellets were rinsed twice with 100 μl cold Tris–HCl buffer and poured into Eppendorf tubes. The membrane suspensions were kept frozen in this latter buffer at-20°C. Lipids were extracted from the membrane suspensions and determined as described for erythrocytes [11-16].

#### Determination of hepatic HMG-CoA reductase activity

This was determined according to the method of Rao and Ramakrishnan [20] by measuring the hepatic concentrations of HMG-CoA and mevalonate. The ratio of HMG-CoA to mevalonate is taken as an index of the activity of HMG-CoA reductase. An increase in this ratio indicates inhibition of cholesterogenesis while a decrease indicates enhanced cholesterogenesis.

### Statistical analysis

Data are expressed as mean ± S.E.M. One way analysis of variance (ANOVA) followed by Duncan Multiple Range Test was used to analyse the results with p < 0.05 considered significant.

## Results

**Figure 2:**
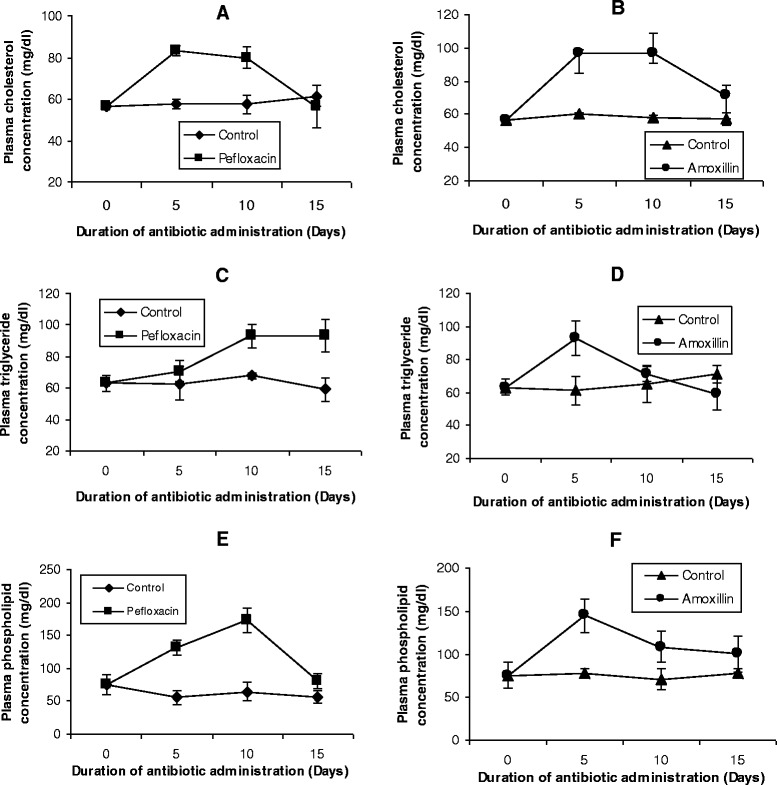
**Effects of pefloxacin (A,C,E) and amoxillin (B,D,F) on plasma lipid profiles of the animals.** Each point represents M±SEM of 5 animals.

**Figure 3:**
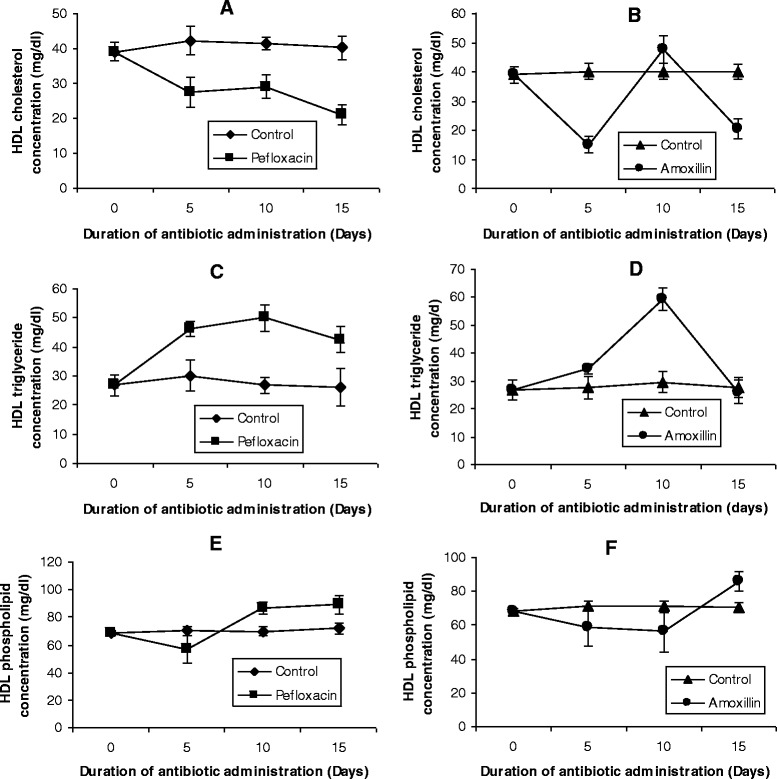
**Effects of pefloxacin (A, C, E) and amoxillin (B, D, F) on HDL lipid profiles of the animals.** Each point represents M±SEM of 5 animals.

**Figure 4:**
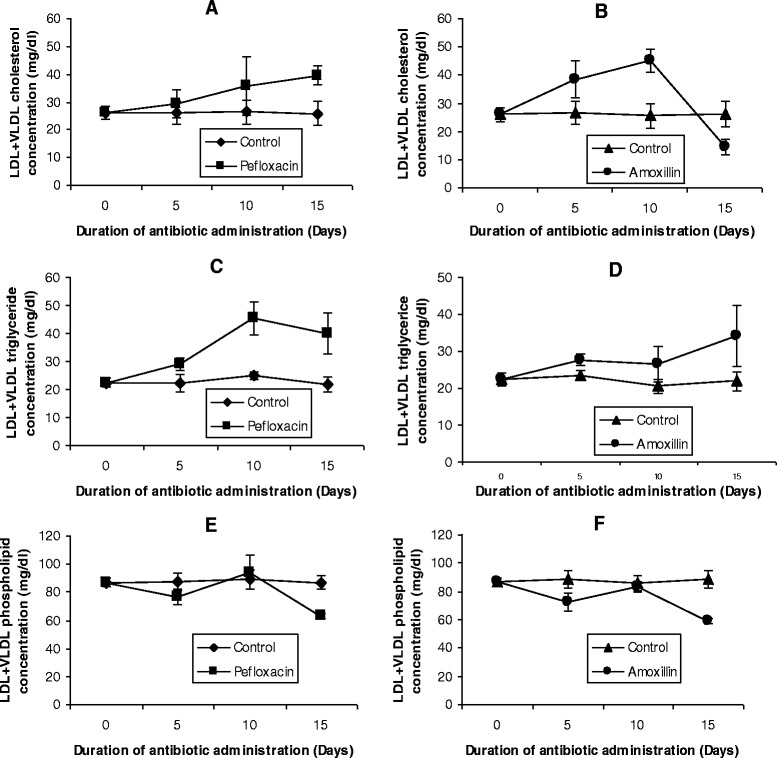
**Effects of pefloxacin (A,C,E) and amoxillin (B,D,F) on VLDL+LDL lipid profiles of the animals.** Each point represents M±SEM of 5 animals.

Figures 2, 3 and 4 depict the effects of pefloxacin and amoxillin on plasma (Figure 2), HDL (Figure 3) and LDL+VLDL (Figure 4) lipid profiles of the animals. Administration of the two antibiotics resulted in various degrees of dyslipidemia in these compartments. In the plasma, administration of the two antibiotics resulted in a significant increase (p < 0.05) in the concentrations of all the lipids. Comparatively, the dyslipidemia was more pronounced in pefloxacin-treated animals when compared with their amoxillin counterparts. Five days after the antibiotics were discontinued, the increase was still sustained, although triglyceride concentration returned to control values in amoxillin-treated animals.

After 5 days of antibiotic treatment, HDL cholesterol decreased by 35% in pefloxacin-treated animals (Figure 3A) and 63% in amoxillin-treated animals (Figure 3B) whereas phospholipid concentrations of HDL decreased by 19% (pefloxacin) (Figure 3E) and 18% (amoxillin) (Figure 3F) respectively. While the decrease in HDL cholesterol was sustained till the end of the experiment (with exception of amoxillin-treated animals on day 10 when a significant increase was observed), there was a rebound to higher HDL phospholipid concentrations in pefloxacin-treated animals on day 10 and 5 days after discontinuing the antibiotic whereas this phospholipidosis was observed in amoxillin-treated animals 5 days after discontinuing the antibiotic.

In contrast to cholesterol and phospholipids, 5 days of antibiotic treatment resulted in a significant increase (p < 0.05) in HDL triglyceride concentrations (Figures 3C and D). While this increase amounted to 53% in pefloxacin-treated animals (Figure 3C), it was 24% in amoxillin-treated animals (Figure 3D). During the 10 day antibiotic treatment, the increase in HDL triglyceride was still sustained. This was however reversed 5 days after discontinuing the antibiotics with the triglyceride concentration in rats treated with amoxillin not significantly different (p >0.05) from that of control animals whereas in pefloxacin-treated animals the increase in HDL triglyceride amounted to 63% compared with controls.

In the LDL+VLDL fraction (Figures 4A and B), administration of the two antibiotics also resulted in a significant increase (p < 0.05) in cholesterol concentrations. While this increase was sustained in pefloxacin-treated animals even 5 days after discontinuing the drug, cholesterol concentration in amoxillin-treated animals was just about 50% of control animals 5 days after the antibiotic was withdrawn. The two antibiotics however increased the LDL+VLDL triglyceride throughout the course of the experiment (Figures 4C and D). The phospholipid profile of this fraction however showed unsystematic significant changes with the 5 days of antibiotic administration causing a decrease, followed by an increase on day 10 and a decrease 5 days after antibiotic withdrawal (Figures 4E and F).

**Figure 5:**
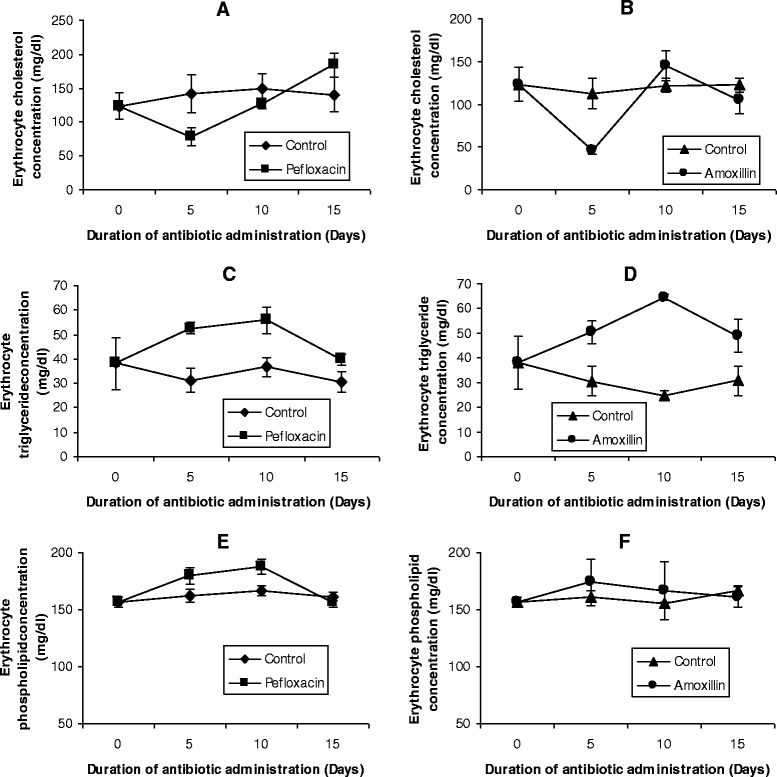
**Effects of pefloxacin (A,C,E) and amoxillin (B,D,F) on erythrocyte lipid profiles of the animals.** Each point represents M±SEM of 5 animals.

**Figure 6:**
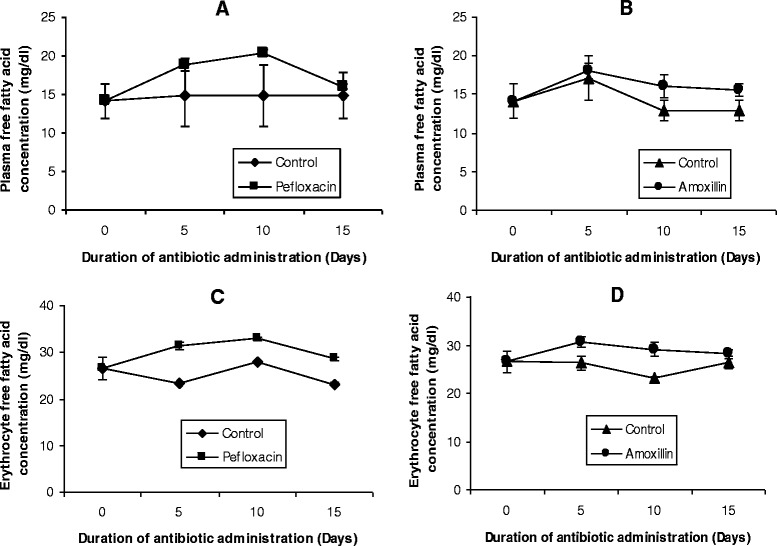
**Effects of pefloxacin (A and C) and amoxillin (B and D) on erythrocyte and plasma free fatty acids of the animals.** Each point represents M±SEM of 5 animals.

The effects of the two antibiotics on the lipid profiles of erythrocyte as well as FFA in plasma and erythrocytes are depicted in Figures 5 and 6. There was a significant decrease (p < 0.05) in erythrocyte cholesterol within 5 days of antibiotic administration (Figures 5A and B). In the pefloxacin-treated animals, a 45% decrease in erythrocyte cholesterol was observed (Figure 5A) while in the amoxillin-treated animals, a 60% decrease in erythrocyte cholesterol was observed (Figure 5B). A rebound to higher cholesterol values was however observed on days 10 and 15 for both antibiotics, although cholesterol values observed in amoxillin-treated animals on day 15 was 15% less than control animals. In contrast to cholesterol, the remaining erythrocyte lipids investigated (triglyceride, phospholipids and free fatty acids) responded to the presence of the two antibiotics with increases in their concentrations. Five days after antibiotic withdrawal, erythrocyte hypertriglyceridemia and phospholipidosis were resolved in pefloxacin-treated animals (Figures 5C and E), whereas the increase observed in erythrocyte free fatty acids was still sustained (Figures 6C and D).

**Figure 7:**
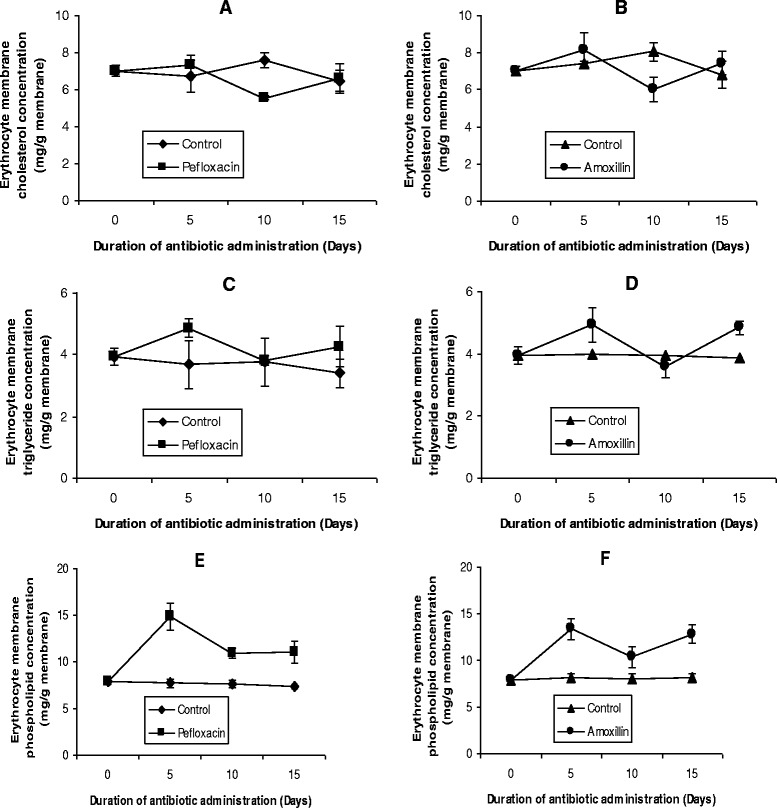
**Effects of pefloxacin (A,C,E) and amoxillin (B,D,F) on erythrocyte membrane lipid profiles of the animals.** Each point represents M±SEM of 5 animals.

The lipid profile of the erythrocyte membrane as presented in Figure 7 also indicates that the two antibiotics induced a phospholipidosis in this compartment (Figures 7E and F) and this was not resolved even 5 days after discontinuing the antibiotics. For cholesterol and triglycerides, the slight changes (both increase and decrease) observed during antibiotic administration had returned to control values 5 days after withdrawing the drugs (Figures 7A-D).

**Figure 8:**
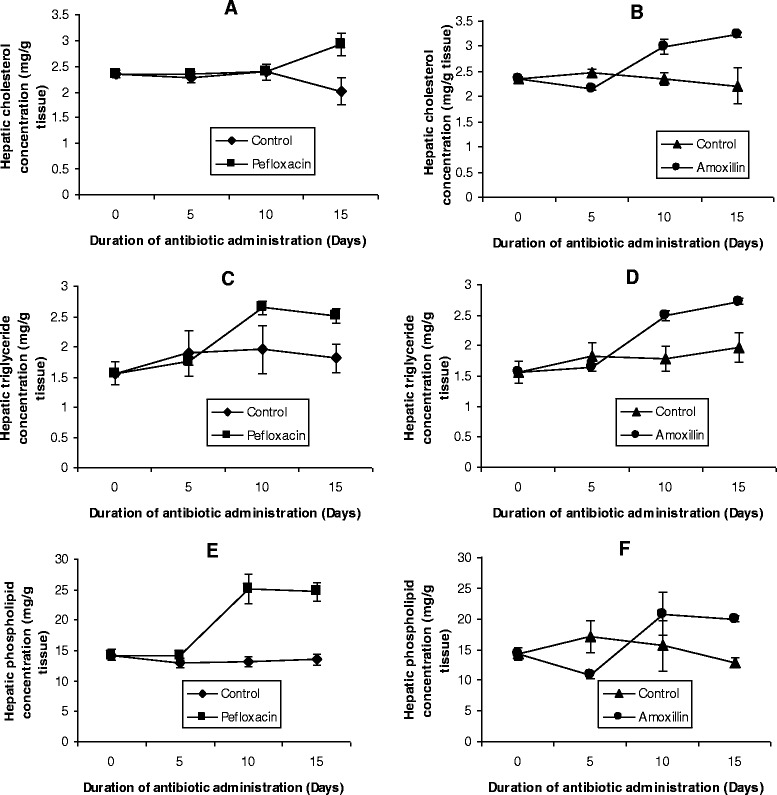
**Effects of pefloxacin (A,C,E) and amoxillin (B,D,F) on hepatic lipid profiles of the animals.** Each point represents M±SEM of 5 animals.

**Figure 9:**
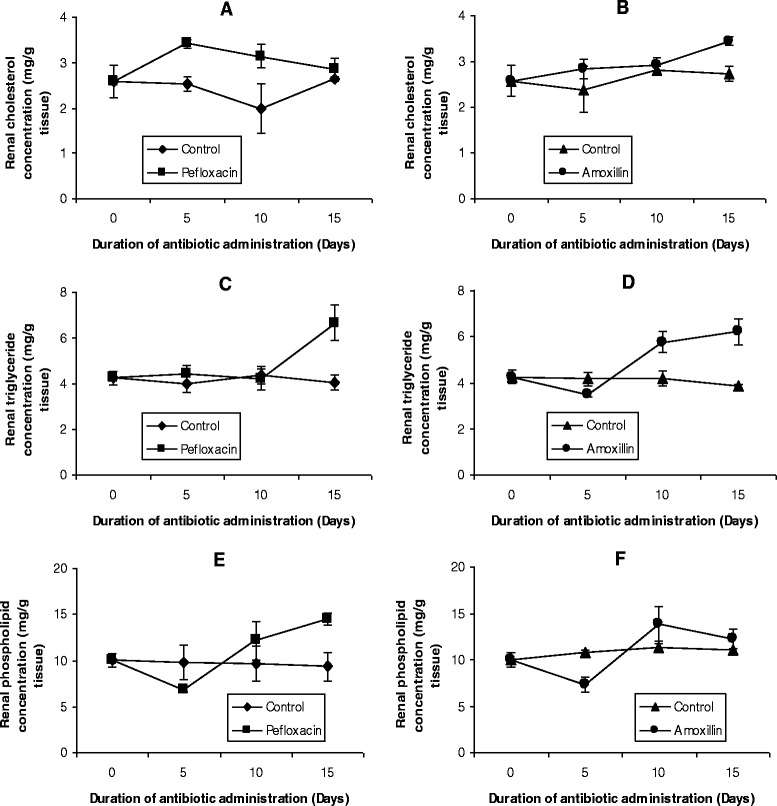
**Effects of pefloxacin (A,C,E) and amoxillin (B,D,F) on renal lipid profiles of the animals.** Each point represents M±SEM of 5 animals.

**Figure 10:**
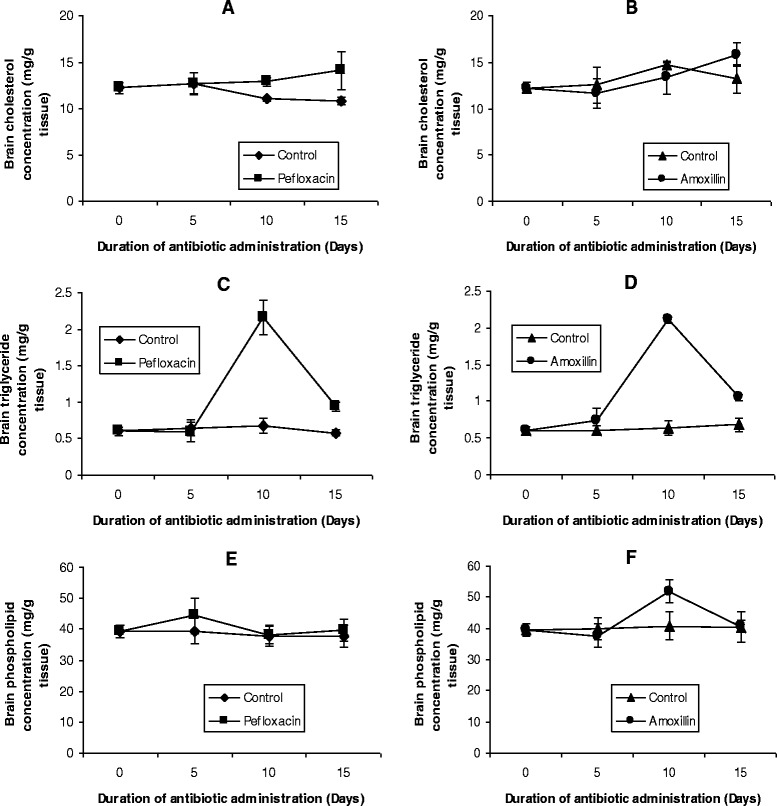
**Effects of pefloxacin (A,C,E) and amoxillin (B,D,F) on brain lipid profiles of the animals.** Each point represents M±SEM of 5 animals.

**Figure 11:**
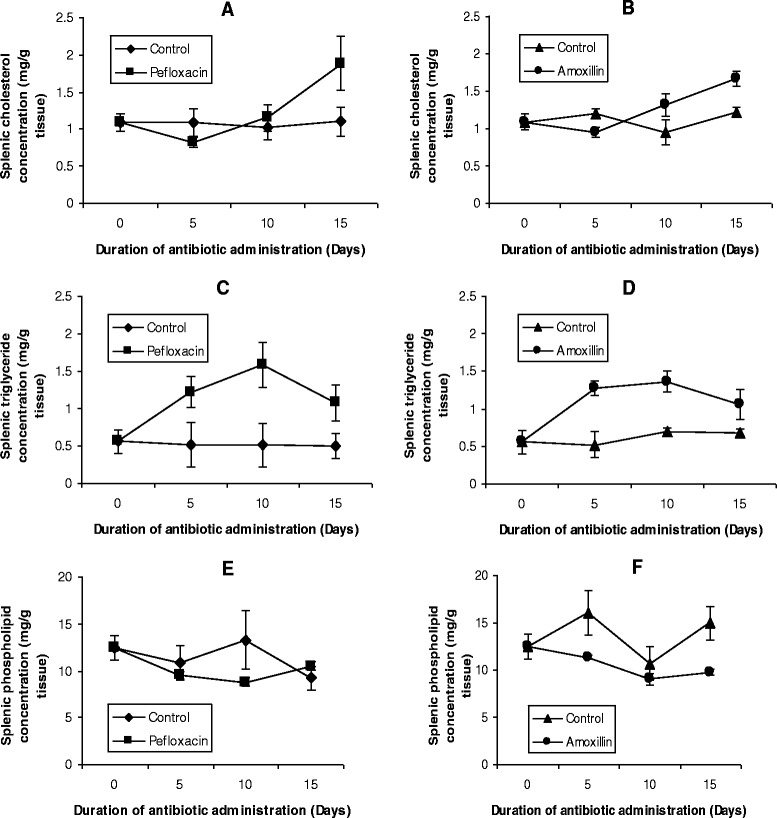
**Effects of pefloxacin (A,C,E) and amoxillin (B,D,F) on splenic lipid profiles of the animals.** Each point represents M±SEM of 5 animals.

**Figure 12:**
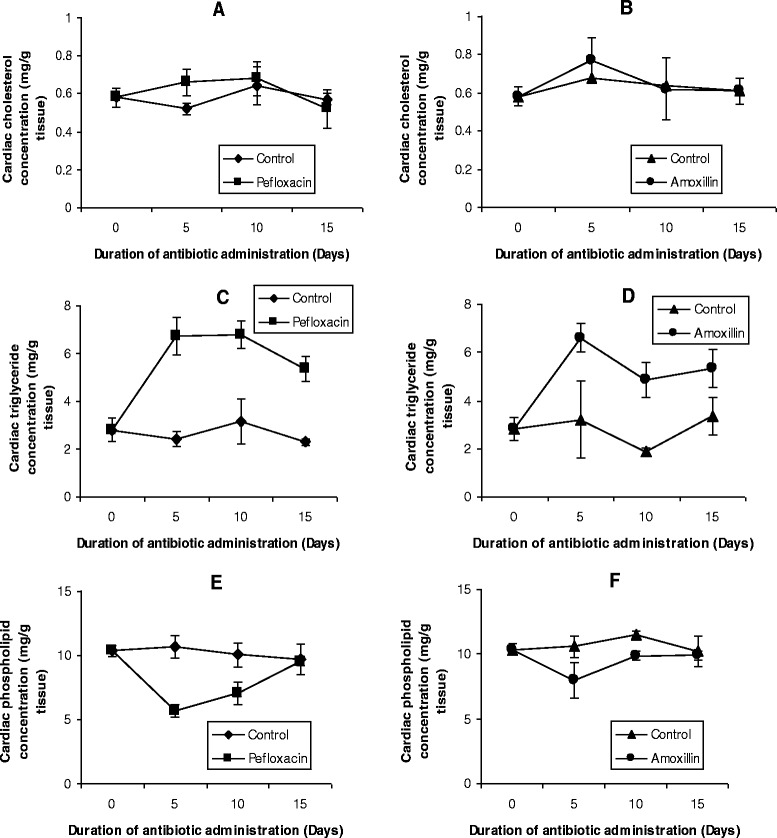
**Effects of pefloxacin (A,C,E) and amoxillin (B,D,F) on cardiac lipid profiles of the animals.** Each point represents M±SEM of 5 animals.

The mean values of the organ lipid profiles are shown in Figures 8, 9, 10, 11 and 12. In all the organs, administration of the antibiotics resulted in a significant increase (p < 0.05) in triglyceride concentrations. The increase ranged from 64% (kidney, Figure 9C; and brain, Figure 10C) to 116% (spleen, Figure 11C) in pefloxacin-treated animals, whereas it ranged from 62% (kidney, Figure 9D) to 94% (spleen, Figure 11C) in amoxillin-treated animals. In contrast however, both cholesterol and phospholipids exhibited different patterns between control and antibiotic-treated animals. In the brain (Figures 10A and B) and heart (Figures 12A and B), antibiotic treatment did not affect cholesterol concentrations. In the spleen, 10 days of antibiotic treatment resulted in the induction of cholesterogenesis (Figures 11A and B), whereas in the kidney induction of cholesterogenesis began with 5 days of antibiotic treatment (Figures 9A and B). In the liver, 10 days of amoxillin treatment resulted in cholesterogenesis (Figure 8B), whereas pefloxacin did not induce cholesterogenesis until 5 days after withdrawing the drug (Figure 8A). In all the organs where cholesterogenesis was observed, the peak of cholesterogenesis was observed 5 days after discontinuing the drugs.

The mean phospholipid concentrations in the organs as depicted in Figures 8, 9, 10, 11 and 12 indicate that antibiotic treatment resulted in a marked hepatic (Figures 8E and F) and renal phospholipidosis (Figures 9E and F) and a transient brain phospholipidosis (Figures 10E and F) whereas splenic (Figures 11E and F) and cardiac (Figures 12E and F) phospholipid concentrations decreased. Hepatic phospholipids increased by 91% (pefloxacin) (Figure 8E) and 55% (amoxillin) (Figure 8F) whereas renal phospholipids increased by 54% (pefloxacin) (Figure 9E) and 22% (amoxillin) (Figure 9F) respectively.

**Figure 13:**
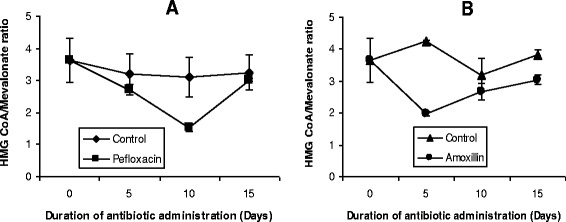
**Effects of pefloxacin (A) and amoxillin (B) on HMG CoA/Mevalonate ratio of the animals.** Each point represents M±SEM of 5 animals.

The ratio of hepatic HMG-CoA/mevalonate as an index of HMG-CoA reductase activity is depicted in Figure 13. Within 5 days of antibiotic treatment, the activity of HMG-CoA reductase increased by 26% (pefloxacin) (Figure 13A) and 46% (amoxillin) (Figure 13B) respectively. While the activity of the enzyme started returning to control values during further administration of amoxillin, administration of pefloxacin caused a further increase in the activity of this enzyme. By the end of the 10 day pefloxacin administration, there was a 51% increase in the activity of the enzyme. Five days after withdrawing both antibiotics, HMG-CoA reductase activity was 21% higher than control animals in amoxillin-treated animals whereas it returned to control values in pefloxacin-treated animals.

## Discussion

Lipid and lipoprotein abnormalities have been shown to play a major role in the pathogenesis and progression of various disease conditions [21,22]. More recently, dyslipidemia, most especially phospholipidosis (a condition in which there is an excessive intracellular accumulation of phospholipids), has received considerable attention in the drug industry. Over 250 chemicals capable of inducing a phospholipidosis in cells under conditions of *in vivo* administration or *ex vivo* incubation have been identified [23-25]. Among them are anti-malarials like chloroquine, anti-microbials like gentamicin and anti-psychotics like haloperidol [23-25]. Amiodarone, an anti-arrhythmic drug, has also been shown to induce phospholipidosis [23-25]. Because the functional consequences and clinical implications of the presence of this condition on cellular or tissue function during drug administration are not completely understood, phospholipidosis is presently becoming a confounding factor not only in new drug candidates, but also in assessing the safety of numerous marketed drugs. Whether amoxillin (an extended-spectrum penicillin) or pefloxacin (a third generation fluoroquinolone) induces any dyslipidemia, was the basis of the present study.

The results of this study demonstrate that amoxillin and pefloxacin at therapeutic doses, induced various degrees of compartment-specific dyslipidemia in the animals. While plasma and erythrocyte dyslipidemia were characterised by up-regulation of the concentrations of the major lipids (cholesterol, triglycerides, phospholipids and free fatty acids {FFA}), hepatic and renal dyslipidemia were characterised by cholesterogenesis and phospholipidosis whereas splenic dyslipidemia was characterised by cholesterogenesis and decreased phospholipid concentrations. At the membrane level, phospholipidosis alone was the hallmark of dyslipidemia in the erythrocyte membrane while lipoprotein abnormalities were reflected as down-regulation of HDL cholesterol and up-regulation of HDL triglycerides and phospholipids as well as up-regulation of LDL + VLDL cholesterol and triglycerides. Furthermore, the two antibiotics increased triglyceride levels in all the compartments in addition to increasing the activity of hepatic HMG-CoA reductase. While the erythrocyte phospholipidosis was resolved 5 days after withdrawing the drugs, dyslipidemia observed in other compartments was still not resolved after withdrawing the antibiotics.

In order to avoid equilibrium, the organism must maintain physiological levels of metabolites in its diverse metabolic activities. Any alteration in these levels might spell serious physiological consequences for the organism. Under physiological conditions, fat reserves of the mammalian body are stored as droplets of triglycerides in the adipose tissue. This triglyceride is then hydrolysed to FFA and glycerol with subsequent mobilisation of FFA into the plasma. This hydrolysis prior to release of FFA occurs within the adipose tissue and is catalysed by a triglyceride lipase distinct from lipoprotein lipase which occurs outside the adipose tissue cell [26]. Release of FFA into the plasma is followed by FFA uptake into tissues (including liver, heart, kidney, muscle, lung, testis and adipose tissue), but not readily by the brain [27,28], where they are oxidised or used in the synthesis of triglycerides in that tissue [29,30]. In this study, we observed a steady increase in plasma FFA in antibiotic-treated animals. This suggests (an) antibiotic-induced activation of triglyceride lipase resulting in increased triglyceride hydrolysis in the adipose tissue and subsequent increased mobilisation of the FFA into the plasma. The physiological consequences of this elevated plasma FFA could be viewed from the metabolic roles of FFA. While this elevated plasma FFA should provide an immediate substrate for triglyceride synthesis as well as the source of available fuel for the tissues and also the necessary signal for tissues to oxidise them [29,30], data in Figures 8, 9, 10, 11 and 12 indicate that while amoxillin and pefloxacin did not inhibit the uptake of the FFA by the tissues (they might be said to even promote uptake of FFA), a considerable amount of the FFA was directed towards the synthesis of triglycerides in the antibiotic-treated animals, hence the accumulation of triglycerides in the tissues. Data in Figure 8 also indicate that while the liver could be said to have a limited capacity for triglyceride storage, tissues like heart and kidney accumulated triglyceride to about four-fold that of the liver (Figures 8, 9, 10 and 11). This further suggests that both amoxillin and pefloxacin induced a dysfunction of triglyceride degradation resulting from insufficient mitochondrial ß-oxidation of FFA, hence compromising energy production in the tissues of the antibiotic-treated animals. Although the triglyceride content of the brain compared to other lipids in this organ is very small, administration of amoxillin and pefloxacin resulted in a significant increase of this lipid in the brain. Since the brain cannot take up FFA from the plasma, an antibiotic-induced damage to the blood–brain barrier and subsequent uptake of the plasma FFA might lead to the triglyceride constipation observed in the brain.

The lipid composition of erythrocytes is currently becoming a matter of great interest because the etiology of many diseases have been shown to have their root in erythrocyte lipid abnormalities [31]. Studies have shown that the red cell lipid is composed almost entirely of unesterified (free) cholesterol and phospholipid [32]. The minute amounts of cholesterol esters, the roughly equivalent proportion of phosphatidylcholine and phosphatidylethanolamine, as well as the presence of phosphatidylserine, imprint a pattern of erythrocyte lipid clearly different from that of the plasma environment [33]. Despite this rather divergent lipid composition of the erythrocyte and its plasma environment, circulating mature erythrocytes from mammals are limited in their lipid metabolism in a number of respects. Firstly, there is little evidence of de novo synthesis of lipid by the red cell [34,35]. Secondly, *in vivo* and *in vitro* evidences suggest that a major pathway for replacement of red cell lipids is through exchange with plasma lipids [32,36,37]. Compounds like urea, acetone, alcohols and dimethylsulfoxide have been shown to facilitate this exchange [32]. Since the administration of the two antibiotics resulted in increased plasma concentrations of the major lipids, the increase observed in erythrocyte lipids may be attributed to exchange of these lipids between plasma (lipoproteins) and erythrocytes [36,37]. However, since the concentrations of the erythrocyte and plasma lipids did not attain equal values, it is possible that the lipid levels of erythrocytes may be composed of several pools with more and less readily exchangeable molecules and that amoxillin and pefloxacin promote transfer of these lipids from these other pools into the erythrocyte [38,39]. Nikolic and his colleagues [38,40] reported a rapid rate of exchange of lipids between HDL and erythrocytes. In addition, Meurs and her colleagues [41] also reported that HDL cholesterol might be an important factor in the determination of the lifespan of erythrocytes. These studies suggest a close association between lipids in the HDL fraction and erythrocytes. Although our study found an association between HDL and erythrocyte lipids, the correlation did not reach statistical significance (Data not shown). Whether HDL might be one of these other pools cannot be ascertained at present even though the reduced HDL cholesterol observed in the antibiotic-treated animals suggests that reverse cholesterol transport was inhibited by the two antibiotics.

Lipid dynamics of the plasma have been widely studied during various metabolic malfunctions as a result of their involvement in vascular disorders. Cholesterol and triglycerides have been the components of major interest with very little attention being given to plasma phospholipids as well as lipid profiles of tissues in various pathologies. Our results indicate that administration of amoxillin and pefloxacin was associated with hepatic and renal cholesterogenesis and phospholipidosis as well as splenic cholesterogenesis. Data in Figure 2 suggest that enhanced cholesterogenesis may be attributed to amoxillin- and pefloxacin-induced activation of HMG-CoA reductase (the rate-limiting enzyme in cholesterol synthesis [25]. Manipulation of the activity of HMG-CoA reductase through alterations of its quantity (through synthesis or degradation) and/or its intrinsic catalytic efficiency (through phosphorylation-dephosphorylation), appears to be the most important molecular mechanism by which cells regulate sterol production [42-44]. While changes in activity resulting from phosphorylation-dephosphorylation are rapid and oftentimes transient, changes in the quantity of enzyme protein is a relatively slow-acting, long-term control mechanism of enzyme regulation [43]. In most studies in which rats or cultured cells have been subjected to a particular dietary manipulation and drugs for hours or days, changes in HMG-CoA reductase activity were accounted for by the alterations in the quantity of HMG-CoA reductase protein present [42,45-47]. It is reasonable to assume that amoxillin- and pefloxacin-induced increase in the quantity of HMG-CoA reductase might be the mechanism underlying the activation of this enzyme. Consistent with this hypothesis was the observation of a 5 day time lag before cholesterogenesis was induced in the antibiotic-treated animals.

Phospholipidosis is a lipid storage disorder in which abnormal quantities of phospholipids accumulate in various tissues [23-25,48,49]. Xenobiotic drugs and chemicals, as well as hormones, cofactors and other agents, may alter the metabolism of the cell and result in phospholipidosis [23-25,48,49]. The induction time may be a few days to several months depending on the affinity of the agent for susceptible cells [13,23-25,48]. Four major concepts have been proposed for the mechanism of induction of phospholipidosis: (1) inhibition of lysosomal phospholipase activity-this is generally regarded as the primary mechanism of induction, (2) inhibition of lysosomal enzyme transport as a result of down-regulation of genes involved in lysosomal enzyme transport, (3) enhanced phospholipid biosynthesis due to enhanced FFA availability and (4) enhanced cholesterogenesis [25]. Our data indicate that the last two concepts might be involved in the induction of phospholipidosis in tissues by amoxillin and pefloxacin. Although the activity of phospholipase was not determined in this study, an inhibition of this enzyme by amoxillin and pefloxacin cannot be ruled out [23]. Joshi and his colleagues [49] also proposed that the xenobiotic could bind to phospholipids making the xenobiotic-phospholipid complex resistant to degradation by phospholipases. This mechanism might probably explain the phospholipidosis observed in the erythrocyte membrane of the antibiotic-treated animals. Since other adverse effects have been reported for the two antimicrobials, induction of cholesterogenesis and phospholipidosis might represent additional adverse effects of amoxillin and pefloxacin.

In conclusion, the results of this study indicate that amoxillin and pefloxacin induce a plethora of compartment-specific dyslipidemia and these alterations might contribute to the development of phospholipidosis and cholesterogenesis in tissues. These observations raise the question whether these conditions are toxicologically benign processes or a sign that cellular defence mechanisms are being overwhelmed. Further studies going on in this laboratory are directed towards providing insights into these questions.
